# Protective Activity of *Rhizobium leguminosarum* bv. *viciae* Strain 33504-Mat209 against Alfalfa Mosaic Virus Infection in Faba Bean Plants

**DOI:** 10.3390/plants12142658

**Published:** 2023-07-16

**Authors:** Ahmed Abdelkhalek, Shimaa Bashir, Hamada El-Gendi, Toufic Elbeaino, Wafaa M. Abd El-Rahim, Hassan Moawad

**Affiliations:** 1 Plant Protection and Biomolecular Diagnosis Department, ALCRI, City of Scientific Research and Technological Applications, New Borg El Arab City 21934, Egypt; y_basher@yahoo.com; 2 Bioprocess Development Department, Genetic Engineering and Biotechnology Research Institute, City of Scientific Research and Technological Applications, New Borg El Arab City 21934, Egypt; elgendi1981@gmail.com; 3 Istituto Agronomico Mediterraneo di Bari (CIHEAM-IAMB), Via Ceglie 9, Valenzano, 70010 Bari, Italy; 4 Agriculture Microbiology Department, National Research Centre, Cairo 12622, Egypt; wafaa10m@hotmail.com (W.M.A.E.-R.); hassanmoawad@yahoo.com (H.M.)

**Keywords:** faba bean, *Rhizobium leguminosarum* bv. *viciae*, alfalfa mosaic virus, oxidative stress, gene expression, HPLC

## Abstract

The application of *Rhizobium* spp., nitrogen-fixing plant growth-promoting rhizobacteria, as biocontrol agents to enhance systemic disease resistance against plant viral infections is a promising approach towards achieving sustainable and eco-friendly agriculture. However, their potential as antivirals and biocontrol agents is less studied. Herein, the capability of *Rhizobium leguminosarum* bv. *viciae* strain 33504-Mat209 was evaluated to promote plant growth and enhance faba bean systemic resistance against alfalfa mosaic virus (AMV) infection. Under greenhouse conditions, the soil inoculation with 3504-Mat209 resulted in notable improvements in growth and an increase in chlorophyll content. This led to a marked decrease in the disease incidence, severity, and viral accumulation level by 48, 74, and 87%, respectively. The protective effect of 33504-Mat209 was linked to significant decreases in non-enzymatic oxidative stress indicators, specifically H_2_O_2_ and MDA. Additionally, there were significant increases in the activity of reactive oxygen species scavenging enzymes, such as peroxidase (POX) and polyphenol oxidase (PPO), compared to the virus treatment. The elevated transcript levels of polyphenolic pathway genes (*C_4_H*, *HCT*, *C_3_H*, and *CHS*) and pathogenesis-related protein-1 were also observed. Out of 18 detected compounds, HPLC analysis revealed that 33504-Mat209-treated plants increased the accumulation of several compounds, such as gallic acid, chlorogenic acid, catechin, pyrocatechol, daidzein, quercetin, and cinnamic acid. Therefore, the ability of 33504-Mat209 to promote plant growth and induce systemic resistance against AMV infection has implications for utilizing 33504-Mat209 as a fertilizer and biocontrol agent. This could potentially introduce a new strategy for safeguarding crops, promoting sustainability, and ensuring environmental safety in the agricultural sector. As far as we know, this is the first study of biological control of AMV mediated by *Rhizobium* spp. in faba bean plants.

## 1. Introduction

Legumes are considered to be the most optimal source of vegetable protein. It is noteworthy that the faba bean (*Vicia faba* L.) possesses an average protein content of 27.6 g per 100 g, surpassing the protein content of the majority of pulses available on the market [1]. In addition, it is the most important legume crop cultivated and produced in Egypt and is widely grown throughout the Mediterranean [2]. Additionally, it performs an ecological function by enhancing soil quality through nitrogen fixation and enhancing the nitrogen and phosphorus nourishment of cereals [3]. Faba bean is known to have significant importance in crop rotation and the mitigation of disease cycles caused by diverse plant pathogens. A variety of abiotic and biotic stresses have negatively impacted the cultivation of faba bean, resulting in a decline in crop yield and a decrease in the global area under cultivation of bean plants from 5 million hectares in 1965 to 2.4 million hectares in 2016 [4]. Faba bean is vulnerable to about 40 viruses [5,6]. Such viruses are known to significantly reduce the quantity and quality of crop yields [7].

Alfalfa mosaic virus (AMV, genus *Alfamovirus*, family *Bromoviridae*) is commonly regarded as a significant worldwide hazard due to its broad host range and a broad spectrum of natural hosts, encompassing many herbaceous and woody plant species [8]. It can be readily transmitted through sap inoculation and by various aphid species in a non-persistent manner to a subset of 430 plant species belonging to 51 dicotyledonous families [9]. Crop losses caused by plant pathogens, especially plant viruses, are a global problem that threatens food security [10]. The utilization of agrochemicals to control plant viruses has resulted in detrimental effects on the environment, compromised the safety of food products, caused harm to farmers [11], facilitated the emergence of pesticide resistance [12], and caused the elimination of non-target organisms and beneficial plant microbial relationships [13].

Enhancing the systemic immunity of plants represents a sustainable and efficacious strategy for managing viral infections in plants [7]. The utilization of plant growth-promoting rhizobacteria (PGPR) has been found to enhance plant growth in the presence of diverse biotic and abiotic stressors [14,15,16]. The production of bioactive chemicals by PGPRs has the potential to activate numerous plant protection genes against viruses and/or improve nutrient and water acquisition, thereby inducing plant systemic resistance and enhancing plant growth [17,18,19]. The process of biological nitrogen fixation holds great significance in agriculture as it facilitates the production of nitrogen through the symbiotic relationship between legumes and rhizobia. This, in turn, leads to an increase in nitrogen levels in the soil, thereby promoting the growth of plants [20]. Using plant growth-promoting rhizobacteria (PGPR) has been shown to augment plant growth and immunity, increasing resistance to various environmental stresses and infections [21].

Rhizobia, which belongs to the plant growth-promoting rhizobacteria (PGPRs) group, has exhibited a notable capacity for nitrogen fixation in leguminous plants and eliciting plant defense responses. According to reports, manifesting mosaic, chlorotic, and mottling symptoms caused by AMV infection on faba bean leaves led to significant reductions in crop yield and economic losses [9,22]. Conversely, the introduction of rhizobial inoculation was found to significantly increase the plant defense system [7]. The potential of biocontrol agents, specifically antiviral agents, has not been extensively studied. The objective of this study is to assess the efficacy of *Rhizobium leguminosarum* bv. *viciae* strain 33504-Mat209 in enhancing the growth of faba bean and augmenting its immunity against AMV infection for the first time. In addition, this study aims to evaluate non-enzymatic oxidative stress indicators such as hydrogen peroxide (H_2_O_2_) and malondialdehyde (MDA) and reactive oxygen species (ROS) scavenging enzymes, including peroxidase (POX) and polyphenol oxidase (PPO). Furthermore, an assessment will be conducted on the manifestation of four polyphenolic pathway genes [cinnamic acid 4-hydroxylase (*C_4_H*), *p*-coumarate 3-hydroxylase (*C_3_H*), hydroxycinnamoyl Co A shikimate hydroxycinnamoyl transferase (*HCT*), and chalcone synthase (*CHS*)] and pathogenesis-related protein-1 in faba bean. Moreover, the identification of the polyphenolic compound profile in the faba bean plant extract will be determined through high-performance liquid chromatography (HPLC) analysis.

## 2. Results

### 2.1. Bacterial Isolation and Identification

Seventeen rhizobia isolates were obtained and subjected to purification from the root nodules of faba beans. The bacterial isolates exhibited Gram-negative characteristics, characterized by their rod-shaped cellular morphology and restricted ability to absorb Congo red. This resulted in the formation of colonies that displayed a pale pink to white coloration on the growth medium. The rhizobial isolate exhibiting the greatest level of symbiotic effectiveness and antiviral activity was selected and subsequently subjected to characterization. Nucleotide sequencing of an amplified 16S rRNA gene identified the investigated bacteria as Rhizobium leguminosarum bv. viciae. The sequence and its description have been sent to GenBank, where they can be found under the name R. leguminosarum bv. viciae strain 33504-Mat209 with the accession number OR121484. NCBI-BLAST and phylogenetic tree analyses indicated that the 33504-Mat209 displayed a significant level of similarity to other strains of R. leguminosarum bv. viciae, with an identity ranging from 97% to 98%. This similarity was determined by comparing the sequences of the 33504-Mat209 strain to the related sequences present in the GenBank database.

### 2.2. The AMV-CP Accumulation Level and Disease Severity Evaluation

**Figure 1:**
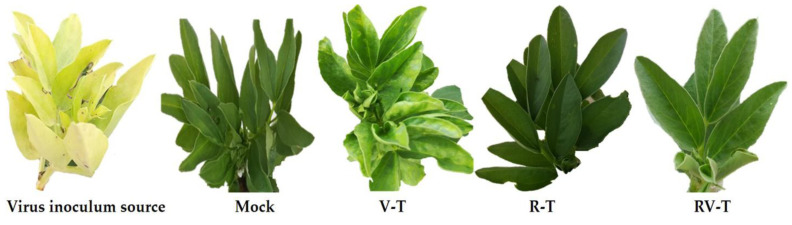
Effect of soil application of *R. leguminosarum* bv. *viciae* strain 33504-Mat209 on developing disease symptoms in faba bean leaves at 16 dpi. The virus inoculation source showed a clear calico characteristic symptom of AMV infection; Mock: healthy control; V-T: faba bean inoculated with AMV only; R-T: faba bean inoculated with 33504-Mat209 only; RV-T: faba bean treated with 33504-Mat209 and then inoculated with AMV.

Under controlled greenhouse conditions, the soil application of 33504-Mat209 to faba bean plants (RV-T) resulted in a significant reduction in disease severity and a decrease in AMV accumulation level when compared to those inoculated with AMV only (V-T). The results showed that the V-T treatment developed characteristic symptoms at 11 dpi, while the application of 33504-Mat209 delayed symptom appearance by five days, with mild symptoms at 16 dpi (Figure 1). On the other hand, clearly visible calico symptoms started to develop at 35 to 40 dpi, similar to those that appeared on AMV inoculum source tissues (Figure 1). No observed symptoms were present in either the Mock or R-T treatment groups.

**Figure 2:**
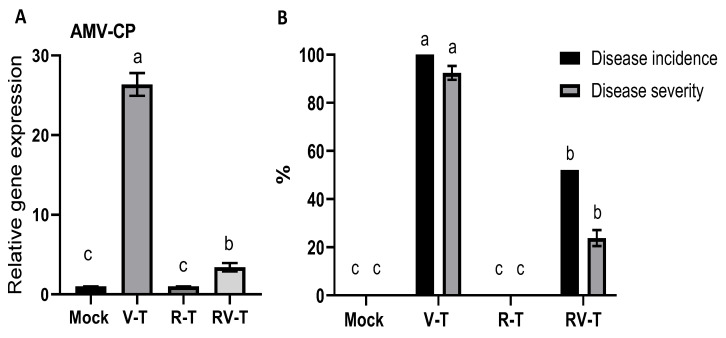
The relative accumulation of AMV coat protein (*AMV-CP*) is represented in (**A**) with associated disease incidence % and disease severity % (**B**) in healthy control (Mock), AMV-infected (V-T), healthy plant-*Rhizobium* treated (R-T), and AMV-infected-treated with *Rhizobium* (RV-T). Statistical significance was indicated alphabetically in each column in ascending order, whereas a > b > c.

Figure 2 shows that the level of *AMV-CP* expression in V-T plants increased by up to 26.4-folds compared to the level in Mock plants at 21 dpi. In the RV-T, the *AMV-CP* expression was 3.42-fold that of Mock plants, which asserted the AMV infection. The reduction in *AMV-CP* accumulation in RV-T represents about 87% of the V-T level, which asserts the ability of 33504-Mat209 to retard viral replication and CP accumulation. On the other hand, the disease incidence and severity results (Figure 2) were in line with the *AMV-CP* accumulation level, whereas both Mock plants ns R-T revealed no signs of AMV infection. On the contrary, the maximum disease incidence and severity were detected in V-T, at about 100 and 92.4%, respectively. Additionally, the RV-T treatment revealed a significant reduction in disease incidence and severity, accounting for 48 and 74%, respectively, compared to the V-T group.

### 2.3. Evaluation of Plants’ Growth Parameters and Total Chlorophyll Contents

Plant growth is severely affected by various viral infections, which could consequently affect the final crop yield. As indicated in Table 1, the AMV infection significantly affected the shoot system length and fresh weight to 29.9 ± 1.5 cm and 6.32 ± 0.8 g, respectively, as compared to healthy plants (Mock group) 37.2 ± 1.3 cm and 7.23 ± 0.9 g, respectively. The reduction in shoot system length and weight represents about 20% and 12.6%, respectively, from Mock plants. Treatment with 33504-Mat209 significantly enhanced the length and weight of the shoot system in RV-T (34.2 ± 1.6 cm and 6.73 ± 1.1 g, respectively) and maximized them in the R-T group (42.3 ± 2.0 cm and 8.14 ± 1.1 g, respectively). The maximum shoot system length and fresh weight in the R-T group represent a 14.3% and 12.6% increase in length and weight, respectively, over those of Mock plants.

In the same direction, the root system was also negatively affected by AMV infection (Table 1). Compared to Mock plants, the length and weight of the root system in the V-T group were reduced by about 40.6% and 28.6%, respectively. Treatment with 33504-Mat209 enhanced the root system length and fresh weight, even over that of Mock in the R-T group (29.2 ± 2.3 cm and 6.76 ± 1.0 g, respectively) and in the RV-T group (24.6 ± 2.1 cm and 6.23 ± 1.1 g, respectively).

Furthermore, the total chlorophyll contents in all groups were evaluated calorimetrically. The results in Table 1 revealed a 29.8% reduction in total chlorophyll contents attributed to AMV infection (V-T) compared to Mock plants (26.20 ± 1.2 units). The R-T group revealed a 15% increase in the total chlorophyll content (30.81 ± 1.7 units) over Mock plants. Additionally, the 33504-Mat209 treatment was able to retard the adverse effects on total chlorophyll level in RV-T groups (25.65 ± 1.6 units), which is nearly the total chlorophyll level in Mock plants.

### 2.4. Evaluation of Oxidative Stress Markers

**Figure 3:**
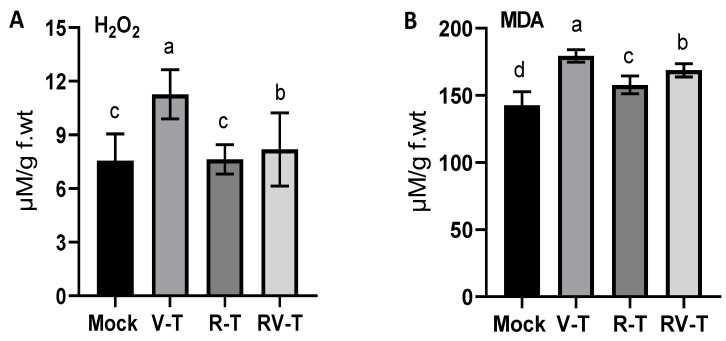
Oxidative stress biomarkers were evaluated in faba bean plants under AMV challenge, including H_2_O_2_ accumulation (**A**) and MDA level (**B**) in healthy control (Mock), AMV-infected (V-T), health plant-*Rhizobium* treated (R-T), and AMV-infected-treated with *Rhizobium* (RV-T). Statistical significance was indicated alphabetically above each histogram in ascending order, whereas a > b > c > d.

Oxidation stress is a hallmark of viral infection, which is usually associated with the accumulation of free radical species and could be detected through several oxidation stress markers. In the present study, H_2_O_2_ level (Figure 3A) was considerably increased in faba bean plants infected with AMV (about 50%), as indicated in the V-T group (11.27 ± 1.38 µM/g. f.wt) compared to Mock plants (7.55 ± 1.51 µM/g. f.wt). Treatment with 33504-Mat209 insignificantly affected the H_2_O_2_ accumulation as indicated in the R-T group (7.63 ± 0.83 µM/g. f.wt) compared to the control. On the contrary, the AMV-infected plants revealed a significantly lower titer of H_2_O_2_ accumulation when treated with 33504-Mat209 (8.19 ± 2.04 µM/g. f.wt) compared to the V-T group. Though the H_2_O_2_ level is slightly higher than in Mock plants (about 8.5%), the H_2_O_2_ reduction in RV-T represents about 38% of the reduction in the V-T group.

Furthermore, lipid peroxidation is another important oxidative stress marker that could be evaluated through the MDA level. The Figure 3B results indicated about a 26% increase in the MDA level in the V-T group (179.38 ± 4.69 µM/g. f.wt) compared to Mock plants (142.6 ± 10.14 µM/g. f.wt). Treatment of the faba bean seeds with *Rhizobium* slightly enhanced the MDA accumulation by about 10% and 18.3% in R-T and RV-T, respectively, compared to Mock plants. However, the MDA accumulation was alleviated in RV-T by about 17% compared to V-T (Figure 3B).

### 2.5. Antioxidant Enzymes Evaluation

**Figure 4:**
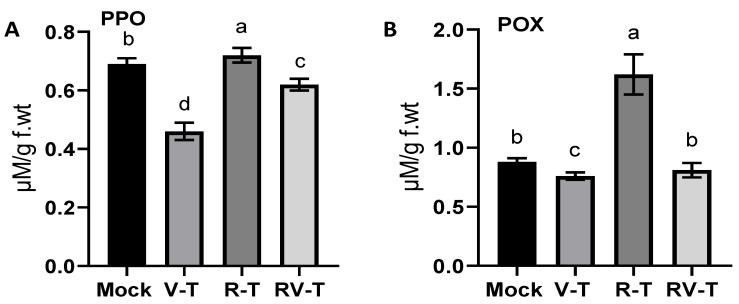
Antioxidant enzyme evaluation in faba bean plant under AMV challenge, including peroxidase (POX) activity (**A**) and polyphenol oxidase (PPO) activity (**B**) in healthy control (Mock), AMV-infected (V-T), healthy plant-*Rhizobium* treated (R-T), and AMV-infected-treated with 33504-Mat209 (RV-T). Statistical significance was indicated alphabetically above the histogram in ascending order, whereas a > b > c > d.

The antioxidant activity of two antioxidant enzymes was evaluated in all groups. As a result of AMV infection, the POX enzyme activity was decreased (about 15.8%) in the V-T group to 0.76 ± 0.03 µM/ g. f.wt compared to 0.88 ± 0.03 µM/g. f.wt in the Mock plants (Figure 4A). In the R-T group, the POX level sharply increased to 1.62 ± 0.17 µM/g. f.wt, representing 1.8- and 2.13-fold increases compared to the Mock and V-T groups, respectively. Under the AMV challenge, the *Rhizobium* treatment slightly enhanced the POX level to 0.81 ± 0.06 µM/g. f.wt, which is still below the POX level in Mock plants.

Furthermore, the PPO activity was also evaluated in all groups. As shown in Figure 4B, the PPO level was significantly reduced in V-T to 0.46 ± 0.01 µM/g. f.wt, which represents about a 50% reduction from the enzyme level in Mock plants (0.69 ± 0.02 µM/g. f.wt). Treatment with 33504-Mat209 enhanced the PPO level in the R-T group to 0.72 ± 0.01 µM/g. f.wt (about 4%) compared to Mock plants, whereas in RV-T, the PPO level was 0.62 ± 0.02 µM/g. f.wt. However, the PPO level was lower in RV-T compared to Mock plants. The 33504-Mat209 treatment succeeded in enhancing enzyme levels by about 56.5% compared to the V-T group (Figure 4B).

### 2.6. Effect of 33504-Mat209 on the Transcriptional Levels of Defense-Related Genes

**Figure 5:**
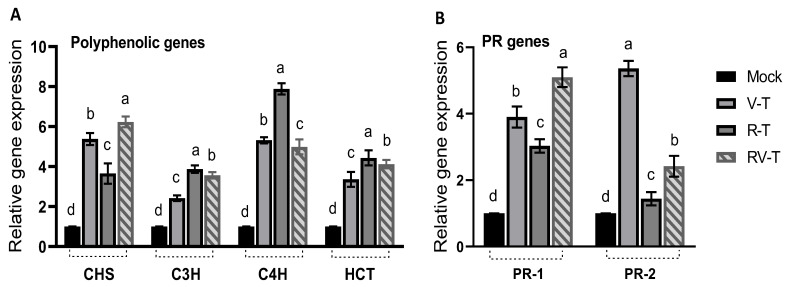
(**A**) the relative expression of polyphenolic genes (*CHS*, *C_3_H*, *C_4_H*, and *HCT*) and (**B**) pathogenesis-related genes (*PR-1* and *PR-2*) of healthy control (Mock), AMV-infected (V-T), healthy plant-*Rhizobium* treated (R-T), and AMV-infected-treated with *Rhizobium* (RV-T). Differences between groups were determined using a one-way analysis of variance (ANOVA) and Tukey’s HSD test at the *p* ≤ 0.05 significance level in the CoStat statistical package. Statistical significance was indicated alphabetically above the histogram in ascending order, whereas a > b > c > d.

Overexpression of several polyphenolic compounds is a known mechanism for plant cells under many biotic and abiotic stresses. The results (Figure 5A) indicated significant upregulation of the *CHS* gene in V-T up to 5.4-folds in Mock plants. Treatment with 33504-Mat209 for healthy faba bean plants (R-T group) increased the *CHS* expression of Mock plants by 3.7-folds, whereas in RV-T, the *CHS* expression was maximized by 6.3-folds compared to Mock plants. The second gene, *C_3_H*, was also activated in V-T by about 2.4 folds compared to Mock plants. The R-T was superior in upregulating the *C_3_H* gene (3.87-fold increase), followed by the VR-T (3.56-fold increase) compared to Mock plants. The third polyphenolic gene evaluated was *C_4_H*, which surged upon AMV to 5.3-folds of the Mock plant level, as indicated in the V-T group (Figure 5A). Similarly, the R-T revealed that the maximum *C_4_H* upregulation accounted for about 7.8-folds of Mock plants. On the contrary, the RV-T group revealed insignificant variation in *C_4_H* gene expression (5-fold of Mock plants) compared to the V-T groups. The last gene evaluated was *HCT*, which was also enhanced in the V-T group by 3.4 folds in Mock plants. Treatment with 33504-Mat209 significantly enhanced gene expression to 4.43 and 4.12 folds in the R-T and RV-T groups, respectively, compared to Mock plants.

On the other hand, two PR gene expressions were evaluated in all groups. As shown in the results (Figure 5B), the *PR-1* gene expression was significantly upregulated in V-T to 3.9-fold that of Mock plants. In the R-T, the gene expression was slightly lower than that of the V-T group but enhanced to 3-fold that of Mock plants. The RV-T was superior in enhancing *PR-1* expression and accounted for 5.1-fold increases compared to Mock plants, about 30% higher than the V-T group. The second PR gene evaluated was *PR-2*, which significantly surged in V-T to 5.4-fold that of Mock plants. Compared to Mock plants, the R-T and RV-T enhanced the *PR-2* gene expression by 1.44 and 2.42 folds, respectively.

### 2.7. Polyphenolic Compounds Analysis through HPLC

**Figure 6:**
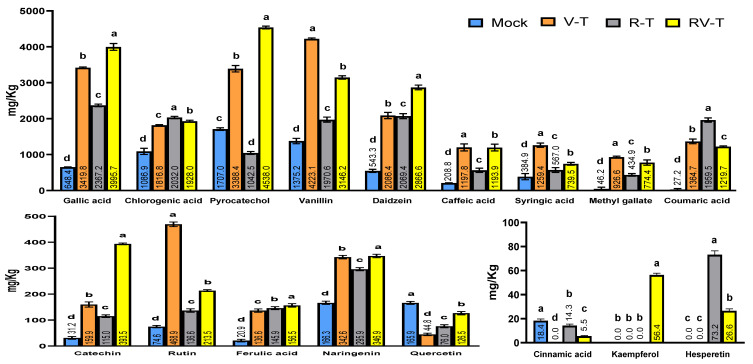
The variation in polyphenolic compound concentration (mg/kg) in the healthy plants (Mock), AMV-infected (V-T), healthy plant-*Rhizobium* treated (R-T), and AMV-infected-treated with *Rhizobium* (RV-T) as evaluated through high-performance liquid chromatography (HPLC) analysis. Differences between groups were determined using a one-way analysis of variance (ANOVA) and Tukey’s HSD test at the *p* ≤ 0.05 significance level in the CoStat statistical package. Statistical significance was indicated alphabetically above the histogram in ascending order, whereas a > b > c > d.

The analysis of changes in phytochemical constituents within different groups of faba beans was conducted by examining leaf samples from four treatments using HPLC. The findings (Figure 6) showed significant differences in the polyphenolic constituents between treatment groups. Out of the 19 evaluated compounds, only apigenin was absent in all groups. The total amounts of the 18 polyphenolic compounds identified were 6824.95, 20,835.58, 13,859.44, and 21,727.34 mg/kg in the Mock, V-T, R-T, and RV-T groups, respectively. The results asserted the direct effect of AMV infection and 33504-Mat209 treatment on final polyphenolic contents compared to Mock plants. With an availability titer of more than 1000 mg/kg, the main chemicals found were chlorogenic acid, pyrocatechol, and vanillin, contrary to cinnamic acid, kaempferol, and hesperetin, which were the least detected compounds (lower than 100 mg/kg). Interestingly, cinnamic acid was completely absent in the V-T group, while it was detected in R-T and RV-T at levels of 14.27 and 5.53 mg/kg, respectively. Additionally, the hesperetin compound was found exclusively in R-T and RV-T groups, with concentrations of 73.22 and 26.6 mg/kg, respectively. n contrast, the compound was absent in Mock and V-T treatments. 

## 3. Discussion

The faba bean (*Vicia faba* L.) is a significant legume with high protein levels and essential amino acids in its seeds, making it a valuable source of nutrition for both humans and animals. However, it also contains anti-nutritional factors, including vicine, convincing, and tannin [23]. Many commercially significant plant groups, such as *Solanaceae* and *Leguminosae*, are susceptible to AMV infection due to their global distribution [24,25]. Such viruses result in significant reductions in agricultural productivity. So, one of the foremost considerations in ensuring food security is the identification of novel biocontrol agents that possess both environmental safety and the ability to counteract plant virus infections while facilitating nitrogen fixation effectively. In the present study, the protective properties of *R. leguminosarum* bv. *viciae* strain 33504-Mat209 against AMV in faba bean were assessed. The 33504-Mat209 morphological characteristics aligned with the description of *R. leguminosarum*, as outlined in Bergey’s manual of systematic bacteriology [26]. In addition, the morphological findings of 33504-Mat209 were corroborated by conducting sequence analyses on 16S rRNA. GenBank accession number OR121484 represents the sequence after annotation.

Under greenhouse conditions, the application of 33504-Mat209 significantly improved faba bean physiological parameters compared to AMV-infected plants without 33504-Mat209 treatment (V-T). Compared to the V-T treatment, the RV-T (33504-Mat209 + AMV) treatment significantly increased shoot and root parameters and chlorophyll content. Such results exhibit the characteristics of PGPR for 33504-Mat209, which can be attributed to its direct impact on enhancing nutrient accessibility, generating phytohormones that promote plant growth, and facilitating water absorption [7,14,27]. Notably, the utilization of 33504-Mat209 demonstrated a correlation with a reduction in disease occurrence by as much as 48% and a decrease in severity by up to 74%. Consequently, this led to a postponement of AMV-induced symptoms by five days compared to plants that were not treated. In addition, the noteworthy decrease in the accumulation level of AMV (up to 87%) confirms the protective effect of 33504-Mat209 against AMV infection. Hence, using 33504-Mat209 may potentially enhance the host’s endogenous immune response and induce systemic acquired resistance (SAR), thereby impeding the dissemination of AMV and suppressing its replication. The findings were consistent with the outcomes reported in previous studies [7,27], which demonstrated that the application of *R. leguminosarum* on faba bean plants resulted in a notable reduction in the occurrence of BYMV infection compared to untreated plants. Increasing levels of H_2_O_2_ and MDA, which are markers of oxidative stress, are in line with the typical characteristic of plant viral infections, wherein there is a concurrent presence of a significant amount of ROS [28,29]. The utilization of 33504-Mat209 exhibits a noteworthy reduction in the levels of both oxidative stress indicators. The maintenance of cell membrane integrity and stability was associated with inhibiting enzymes that generate oxidative stress [30,31]. The results indicate that 33504-Mat209 is effective in reducing oxidative stress in plants infected with a virus, as evidenced by the significant reduction in MDA and H_2_O_2_ levels. Similarly, elevated levels of antioxidant enzymes like POX and PPO in virus-infected tomato plant leaves strengthened cell walls, limiting tissue oxidation and pathogen penetration [32,33]. The elevated levels of both enzymes suggest a potential significance of these enzymes in the detoxification of ROS in faba bean plants after 33504-Mat209 treatment [34].

Numerous studies have shown that activating PR proteins is crucial for SAR and can significantly mitigate pathogen proliferation, multiplication, and spread [35,36]. Compared to mock treatments, all three treatments significantly induced the transcriptional level of *PR-1*, with a maximum level (5.1-fold) detected in RV-T. The phytohormone molecule salicylic acid (SA) is a widely recognized plant signal, and its involvement in activating plant immunity has been extensively studied for over twenty years [37,38]. Moreover, many research studies have demonstrated that *PR-1* serves as a marker gene for SAR and is a forecaster of the initial plant defensive reaction [38,39]. The upregulation of *PR-1* is frequently associated with the buildup of salicylic acid (SA), resulting in the initiation of systemic acquired resistance (SAR). Consequently, we postulate that 33504-Mat209 could generate elicitor metabolites that trigger systemic resistance, stimulating SAR and augmenting the plant’s resistance to viral infections. Concerning the expression of *PR-2*, the findings of our study indicate a noteworthy upregulation of *PR-2* in V-T treatment, exhibiting a relative expression level that is 5.36 times higher than that of the control group. While RV-T treatment resulted in an up-regulation of *PR-2* in plants, the expression level was notably lower compared to AMV infection in the absence of 33504-Mat209 treatments. Apart from their principal function in facilitating viral movement between cells, *PR-2*-encoding β-1,3-glucanases also facilitate intercellular communication and long-range signaling by restricting callose deposition in the vicinity of plasmodesmata [40,41]. Hence, it is plausible that AMV may trigger the activation of *PR-2* to enhance its mobility and proliferation within the cellular structure of plants. This observation aligns with prior investigations, which have reported a significant upregulation of *PR-2* in response to viral infections in various plant species such as potato, Arabidopsis, onion, tobacco, and tomato [35,42,43].

Polyphenolic compounds are significant secondary metabolites that contribute to plant growth and protect against various biotic and abiotic stresses [44]. The major classes of polyphenol compounds can be synthesized through three distinct pathways, namely the phenylpropanoid pathway, the chlorogenic acid pathway, and the flavonoid pathway [45,46]. The cinnamic acid 4-hydroxylase (*C_4_H*) is a principal enzyme in the phenylpropanoid pathway, catalyzing the conversion of cinnamic acid to *p*-coumaric acid. In the current study, the expression of *C_4_H* was overexpressed in V-T, R-T, and RV-T treatments with relative expression levels of 5.31-, 7.88-, and 4.98-fold, respectively, compared to the control. In accordance with the findings of transcriptional levels, the HPLC analysis demonstrated that *p*-coumaric acid exhibited the highest induction and accumulation (1959.5 mg/kg) in the R-T treatment. This was followed by the V-T and RV-T treatments, which recorded amounts of 1364.7 mg/kg and 1219.6 mg/kg, respectively. Consequently, the enhancing transcripts of *C_4_H* in *Rhizobium* and/or virus treatment reflect its role in defense against viral infection and suggest that 33504-Mat209 might be used to combat AMV infections as a biocontrol agent activating polyphenolic secondary metabolite biosynthesis in plant tissues [47]. *HCT* is the initial enzyme in the biosynthesis of chlorogenic acid. It helps turn *p*-coumaroyl CoA into shikimate. *C_3_H* then changes shikimate into p-coumaroyl shikimate, which results in chlorogenic acid [48]. Chlorogenic acid is a polyphenolic compound categorized as a phenolic acid that can result from the esterification of caffeic acid and quinic acid. Several reporters stressed its capability to improve plant disease resistance by inhibiting pathogens, including viruses [49,50]. Similar to the *C_4_H* transcript, *HCT* and *C_3_H* genes exhibited an increase in relative expression levels, with a maximum ratio in R-T treatment followed by RV-T and V-T treatment. The upregulation of transcriptional levels of such genes indicates their antiviral function, suggesting that chlorogenic acid can serve as a plant defense mechanism against viral infection besides its role in systemic resistance support. Consistent with the findings, the HPLC analysis showed chlorogenic acid accumulation within plant tissues that may be elicited by SAR and confer heightened resistance to AMV infection [18,35].

Chalcone synthase (*CHS*) is the primary enzyme in the flavonoid pathway that catalyzes the conversion of *p*-coumaroyl CoA into naringenin chalcone, which is subsequently converted to naringenin, the precursor compound necessary for synthesizing most flavonoids [45,46]. The results demonstrated that AMV infection and/or *Rhizobium* treatments significantly elevated *CHS* expression in faba bean plant tissues, with the greatest expression level (6.23-fold) in RV-T treatment. These results are in agreement with Abdelkhalek [18], who reported the induction of *CHS* in potato tissues upon AMV infection. Overexpression of *CHS* has been shown to cause an increase in the levels of flavonoid and isoflavonoid molecules, which have been shown to have antimicrobial activity against a wide variety of phytopathogens [18,51,52,53]. Among flavonoid compounds detected in HPLC analysis, the highest levels of naringenin compounds were detected in V-T and RV-T treatments, supporting qRT-PCR results. Interestingly, the application of 33504-Mat209 induced the kaempferol compound by 56.38 mg/kg in RV-T treatment only. The absence of this compound in mock, V-T, and R-T treatments suggested that it plays a significant role in plant defense against viruses. Kaempferol is one of the most notable flavonoids that plays a vital role in the auxin-dependent defensive response that halts the propagation of viruses throughout plants [54]. Additionally, gallic acid, vanillin, daidzein, caffeic acid, and ferulic acid were also detected at high levels in all treatments compared to the control. Such compounds were previously reported to have different antimicrobial properties. Based on our results, it can be inferred that 33504-Mat209 has the potential to serve as a viable biocontrol agent for mitigating plant viral infections. However, further investigation is necessary to validate the feasibility of implementing this in practical settings.

## 4. Materials and Methods

### 4.1. Plant Material and Viral Inoculum Source

The Agriculture Research Center in Egypt kindly provided a virus-free line of the faba bean (*Vicia faba* L.) cultivar Nobaria 1 that was susceptible to infection by AMV. The strain of alfalfa mosaic virus (AMV) employed in this investigation, KH1 (MN099289), has been previously characterized [25].

### 4.2. Isolation and Characterization of the Most Potent Bacterial Isolate

The bacterial isolate applied during the current work was locally isolated among several other isolates from rhizosphere soil samples collected from faba bean open fields (Alexandria, Egypt). The isolate was selected according to its potent antiviral activity [55] and symbiotic effectiveness with faba bean plants [56]. The 16S rRNA sequencing methodology was applied to identify the selected isolate at the molecular level. The extraction of total genomic DNA was performed using the Genomic DNA Purification Kit (Wiz-ard, Promega Corporation, Madison, WI, USA), following the manufacturer’s instructions. Polymerase chain reaction (PCR) was used to amplify the 16S rRNA gene using the universal primers for 16S rRNA, as shown in Table 2. A computerized DNA sequencer then sequenced the amplified gene. BLASTN was used to look for the closest match between the acquired sequence and the sequences in the GenBank database. The phylogenetic relationship was also depicted by constructing a bootstrap neighbor-joining tree using the MEGA11 program.

### 4.3. Inoculum Preparation and Greenhouse Experimental Design

One hundred milliliters of Yeast Extract Mannitol (YEM) broth medium were infected with a single pure colony of Rhizobium isolate. The microbial culture was incubated at a temperature of 28 °C and agitated at 150 revolutions per minute for 48 h, resulting in a final concentration of 10^9^ colony-forming units per milliliter. Each pot of rhizobia treatments was inoculated with 2 mL of bacterial culture during faba bean seed cultivation. On the other hand, the virus was inoculated on the 20th day of seed germination. Following surface sterilization, the homogeneous faba bean seeds were cultivated in plastic pots measuring 40 cm in diameter and filled with a sterilized, homogeneous blend of clay, sand, and peat moss. The experiment was performed with four treatments. The first treatment (control group, Mock) involved faba bean plants inoculated with a non-inoculated broth medium, followed by viral inoculation buffer. The second treatment (virus treatment group, V-T) involved faba bean plants inoculated with a non-inoculated broth medium, followed by the mechanical inoculation of plants with AMV. The third treatment (Rhizobia treatment group, R-T) involved the inoculation of plants with the *Rhizobium* isolate. The fourth treatment (rhizobia + virus treatment group, RV-T) included plants inoculated with rhizobia culture during cultivation and mechanically inoculated with the virus.

There were five biological replicates (five pots) in each treatment. Each pot contained five faba bean plants. The experimental procedure involved the execution of three technical replicates for each biological replicate. Each biological replicate comprised a cluster of 15 leaves of the faba bean plant, which were gathered from the five plants grown in every pot, with three leaves per plant at 21 days post-viral inoculations (dpi). Per the viral infection, each plant’s two authentic upper leaves were subjected to carborundum dusting (600 mesh) and subsequently mechanically inoculated with AMV [35]. The plants were cultivated in a greenhouse environment free from insects, with a temperature of 28 °C during the day and 16 °C at night and a relative humidity of 70%. The plants were monitored daily for the manifestation of symptoms.

### 4.4. Virus Accumulation Level and Disease Assessment

The relative abundance of the *AMV-CP* gene (Table 1) in plant tissue samples from various treatments was used to determine the viral accumulation level [18]. The level of viral accumulation was determined by comparing the expression of the AMV-CP gene to that of housekeeping genes (*actin* and *EF1-α*). The disease severity (DS) assessment was conducted through visual observation and a rating scale ranging from 0 to 3. The numerical values assigned to the observed symptoms are as follows: 0 denotes the absence of any symptoms; 1 denotes the presence of leaf necrosis; 2 denotes the manifestation of chlorotic and mild mosaic symptoms; and 3 denotes the occurrence of stem necrosis and malformation symptoms. The DS values were expressed as the relative percent that was deducted from the following equation:

$$\mathrm{DS}\;(\%)=\frac{Disease\;scale\times Number\;of\;plants\;per\;scale}{Total\;number\;of\;plants\times Highest\;disease\;scale}\times 100$$

### 4.5. Evaluation of Plants’ Growth Parameters

The growth parameters, including shoot and root system weights (g) and length (cm) of faba bean plants under the AMV challenge, were evaluated in each group at the end of the cultivation experiment (21 dpi).

### 4.6. Total Chlorophyll Determination

The total chlorophyll contents in all groups were expressed by evaluating the total contents of chlorophyll A (Chlr-a) and chlorophyll B (Chlr-b) calorimetrically [57]. Briefly, leaf extracts from all groups were prepared separately after 20 dpi by grinding 1 g of leaves in 10 mL acetone (80%). The mixture was then incubated overnight at 4 °C before centrifugation at 5000 rpm for 10 min. Afterward, using a blank solution of acetone (80%), the absorbance was measured at 645 nm (Chlr-a) and 663 (Chlr-b) through a UV–Visible spectrophotometer (Beckman Coulter Inc., Brea, CA, USA). The total chlorophyll contents (mg/mL) were derived by adding Chlr-a + Chlr-b and measured through the following equations: Chlr-a concentration (mg/mL) = (11.64 × A_663_ nm) − (2.16 × A_645_ nm), whereas Chlr-b concentration (mg/mL) = (20.97 × A_645_ nm) − (3.94 × A_663_ nm).

### 4.7. Determination of Oxidation Stress Conditions (H_2_O_2_ and MDA)

Two biomarkers were evaluated in all groups to elucidate the oxidative stress conditions, including the accumulation of hydrogen peroxide (H_2_O_2_) and lipid peroxidation (MDA). The H_2_O_2_ accumulation was evaluated using the potassium iodide modified method [58]: one g of air-dried leaves was homogenized by 10 mL trichloroacetic acid (0.1% TCA) to extract the H_2_O_2_ contents. A total of 0.5 mL of clear plant extract, 0.5 mL of potassium phosphate buffer (10 mM, pH 7.0), and 1 mL of potassium iodide (1 M) were added under a gentle vortex for 5 min and then measured at 390 nm. The H_2_O_2_ concentrations (µM/g f.wt.) were deducted from a standard curve of H_2_O_2_ under the same assay conditions. The lipid peroxidation level was also evaluated in all groups as an oxidative stress biomarker by determining the malondialdehyde (MDA) titer. According to Heath and Packer’s [59] method, the color developing through MDA reaction with thiobarbituric acid (TBA) solution (0.5% in 20% trichloroacetic acid) is usually evaluated at 600 nm and expressed as MDA concentration (µM/g of fresh weight).

### 4.8. Antioxidant Enzyme Evaluation

#### 4.8.1. Peroxidase Activity (POX)

The POX activity was assessed in all groups based on Angelini et al.’s colorimetric method [60]. First, the clear plant extract was prepared separately from all group leaves by grinding 5 g of fresh upper leaves of 5 plants (2 leaves/plant) in 20 mL of 100 mM phosphate buffer pH 7.0, then centrifuged at 10,000 rpm for 10 min. The POX activity was evaluated in a final reaction volume of 1200 µL by adding 80 µL of crude plant extract to 500 µL of 5 mM guaiacol and 120 µL of 1 mM hydrogen peroxide. The reaction was then incubated for 10 min at 30 °C, after which the absorbance at 480 nm was measured.

#### 4.8.2. Polyphenol Oxidase Activity (PPO)

The PPO activity in all treatment groups was evaluated through the quinone oxidation method, according to [61]. The reaction mixture included 1 mL of 50 mM quinone (prepared in Tris–HCl buffer, 100 mM, pH 6.0), and 0.5 mL plant leaves extract was incubated at room temperature for 30 min. Afterward, the absorbance was measured spectrophotometrically at 420 nm, whereas the increase in reaction absorbance by 0.001 represents one unit of PPO activity (µM/g of fresh weight).

### 4.9. Evaluation of the Relative Expression of Some Defense-Related Genes

The relative gene expression of some polyphenolic and pathogenesis-related (PR) genes was evaluated through the qRT-PCR approach [35,62]. Two PR genes (*PR-1* and *PR-2*) and four polyphenolic genes (*CHS*, *C_3_H*, *C_4_H*, and *HCT*) were evaluated. RNeasy plant mini kit (QIAGEN, Germany) was applied to extract the total RNA from each group using plant leaves as an RNA source. The RNA concentration and purity were determined by measuring the absorbance at 260 and 280 nanometers (A_260_/A_280_) using a SPECTRO Star Nano instrument (BMG Labtech, Ortenberg, Germany). Additionally, RNA integrity was evaluated based on the appearance of 28S and 18S rRNA bands after electrophoresis on 1.2% agarose gels. Two micrograms of DNase I-treated RNA were applied in the next step as a template for cDNA synthesis using the Super-Script II enzyme (Invitrogen, United States) as described previously [62]. The two housekeeping genes *actin* and *EF1-α* (Table 2) were employed as reference genes in qRT-PCR. The qRT-PCR reaction was carried out following the directions provided by the manufacturer of the SYBR Green PCR Master Mix, as previously described [63]. After conducting 40 cycles, melting curves were acquired in order to eliminate any non-specific products. The quantitative polymerase chain reaction (qPCR) efficiency was assessed for each gene and ranged from 93% to 100% for all genes. The investigation of the melting curve within the temperature range of 55 to 95 °C revealed a singular amplified product for all genes under examination. The algorithm proposed by Livak and Schmittgen [64] was used to precisely measure and quantify the relative expression level of the target gene. Results were shown as fold changes in gene expression (fold change > 1 indicates up-regulation and fold change 1 indicates down-regulation) relative to mock-inoculated faba bean.

### 4.10. Evaluation of Polyphenolic Phytochemicals Contents through HPLC

The HPLC (Agilent 1260 Infinity HPLC series) was used to assess the distinct variation in polyphenolic compounds, including phenolics and flavonoids, across all groups. Concerning this matter, leaf samples were obtained from each group, subjected to air drying, and subsequently extracted with 96% mL ethanol (resulting in a final solid material concentration of 2%) while agitated for 6 h. Subsequently, the sample was subjected to evaporation under reduced pressure until complete dryness was achieved. The resulting residue was then introduced into the HPLC system, equipped with a Zorbax Eclipse Plus C18 column measuring 100 mm × 4.6 mm. The HPLC system employed a mobile phase of 0.2% phosphoric acid, acetonitrile, and methanol. The injection volume used for the sample was 20 mL. The phenolic and flavonoid contents were determined using a VWD detector set at a wavelength of 284 nm, following established protocols for quantifying phenolic compounds. A total of 19 standard phenolic compounds, including apigenin, caffeic acid, catechin, chlorogenic acid, cinnamic acid, coumaric acid, daidzein, ellagic acid, ferulic acid, gallic acid, hesperetin, kaempferol, methyl gallate, naringenin, pyrocatechol, quercetin, rutin, syringic acid, and vanillin, were used as reference compounds for the analysis.

### 4.11. Statistical Significance and Analysis

The experiments were performed at least five times, and the results are shown as the mean (M) with the standard deviation (SD). Data significance was determined by performing an analysis of variance (ANOVA) in the CoStat program and then applying Tukey’s honestly significant differences (H.S.D.) method at a *p*-value of less than 0.05. The study demonstrated a notable dissimilarity among groups, presented in alphabetical order (a > b > c, and so on). Conversely, identical letters indicated trivial differences between two treatments in the same test.

## 5. Conclusions

The soil application of *Rhizobium leguminosarum* bv. *viciae* strain 33504-Mat209 increased growth and total chlorophyll content in greenhouse-grown faba bean plants. This reduced disease incidence, severity, and AMV accumulation by 48, 74, and 87%, respectively. 33504-Mat209 reduced non-enzymatic oxidative stress indicators (H_2_O_2_ and MDA) and increased POX and PPO, as well as expression levels of pathogenesis-related protein-1 and polyphenolic pathway genes (*C_4_H*, *HCT*, *C_3_H*, and *CHS*). HPLC examination showed that 33504-Mat209-treated plants accumulated more gallic acid, chlorogenic acid, catechin, pyrocatechol, daidzein, quercetin, and cinnamic acid than non-treated plants. Thus, the ability of 33504-Mat209 to promote plant growth and enhance systemic resistance to AMV infection will support its use as a fertilizer and biocontrol agent and may lead to a novel approach to crop protection.

## Figures and Tables

**Table 1:** The faba bean plant growth parameters in terms of shoot and root system length (cm) and weight (g) under AMV challenge with total chlorophyll (Chlr-a + Chlr-b) contents (units) in healthy control (Mock), AMV-infected (V-T), health plant-*Rhizobium* treated (R-T), and AMV-infected-treated with *Rhizobium* (RV-T).

Group	Shoot			Root			Total Chlorophyll Content
	Length (cm)	Fresh Weight (g)	Dry Weight (g)	Length (cm)	Fresh Weight (g)	Dry Weight (g)	
**Mock**	37.2 ± 1.3 b	7.23 ± 0.9 b	2.98 ± 0.3 b	20.1 ± 1.5 c	5.89 ± 1.2 c	2.21 ± 0.9 c	26.20 ± 1.2 b
**V-T**	29.9 ± 1.5 d	6.32 ± 0.8 d	2.11 ± 0.5 d	14.3 ± 1.8 d	4.58 ± 1.1 d	1.67 ± 0.9 d	20.18 ± 1.4 d
**R-T**	42.3 ± 2.0 a	8.14 ± 1.1 a	3.16 ± 0.9 a	29.2 ± 2.3 a	6.76 ± 1.0 a	2.97 ± 1.0 a	30.81 ± 1.7 a
**RV-T**	34.2 ± 1.6 c	6.73 ± 1.1 c	2.72 ± 0.4 c	24.6 ± 2.1 b	6.23 ± 1.1 b	2.57 ± 0.8 b	25.65 ± 1.6 c

Note: Statistical significance was indicated alphabetically in each column in ascending order, whereas a > b > c > d.

**Table 2:** The nucleotide sequences of the primers that were employed in this investigation.

Primer Name	Abbreviation	Direction	Nucleotide Sequence
16S ribosomal RNA	16S rRNA	Forward	AGAGTTTGATCCTGGCTCAG
		Reverse	GGTTACCTTGTTACGACTT
Alfalfa mosaic virus-coat protein	*AMV-CP*	Forward	CCATCATGAGTTCTTCACAAAAG
		Reverse	TCGTCACGTCATCAGTGAGAC
Pathogenesis related protein-1	*PR-1*	Forward	GTTCCTCCTTGCCACCTTC
		Reverse	TATGCACCCCCAGCATAGTT
Endoglucanase	*PR-2*	Forward	TATAGCCGTTGGAAACGAAG
		Reverse	CAACTTGCCATCACATTCTG
Cinnamate 4-hydroxylase	*C_4_H*	Forward	CCCAGTTTTTG AAA TTG GCT TCA
		Reverse	GCCCCATTCTAA GCA AGA GAA CAT C
Hydroxycinnamoyl transferase	*HCT*	Forward	TCT CCA ACC CCT TTT AAC GAACC
		Reverse	CAA CTT GTC CTT CTA CCA CAG GGA A
*p*-coumarate 3-hydroxylase	*C_3_H*	Forward	TTG GTG GCT ACG ACA TTC CTA AGG
		Reverse	GGT CTG AAC TCC AAT GGG TTA TTC C
Chalcone synthase	*CHS*	Forward	CAC CGT GGA GGA GTA TCG TAA GGC
		Reverse	TGA TCA ACA CAG TTG GAA GGCG
Actin	*Actin*	Forward	GTTAGCAACTGGGATGACAT
		Reverse	GTTACGACCACTAGCATAGAGTG
Elongation factor 1-alpha	*EF1-α*	Forward	GTGAAGCCCGGTATGCTTGT
		Reverse	CTTGAGATCCTTGACTGCAACATT

## Data Availability

Experimental data supporting the findings of this study are available from the corresponding authors upon request.
